# Assessing the technical efficiency of health posts in rural Guatemala: a data envelopment analysis

**DOI:** 10.3402/gha.v7.23190

**Published:** 2014-01-20

**Authors:** Alison R. Hernández, Miguel San Sebastián

**Affiliations:** Division of Epidemiology and Global Health, Department of Public Health and Clinical Medicine, Umeå University, Umeå, Sweden

**Keywords:** data envelopment analysis, efficiency, Malmquist, primary health care, health services management, Guatemala

## Abstract

**Introduction:**

Strengthening health service delivery to the rural poor is an important means of redressing inequities. Meso-level managers can help enhance efficiency in the utilization of existing resources through the application of practical tools to analyze routinely collected data reflecting inputs and outputs. This study aimed to assess the efficiency and change in productivity of health posts over two years in a rural department of Guatemala.

**Methods:**

Data envelopment analysis was used to measure health posts’ technical efficiency and productivity change for 2008 and 2009. Input/output data were collected from the regional health office of Alta Verapaz for 34 health posts from the 19 districts comprising the health region.

**Results:**

Technical efficiency varied widely across health posts, with mean scores of 0.78 (SD=0.24) and 0.75 (SD=0.21) in 2008 and 2009, respectively. Overall, productivity increased by 4%, though 47% of health posts experienced a decline in productivity. Results were combined on a bivariate plot to identify health posts at the high and low extremes of efficiency, which should be followed up to determine how and why their production processes are operating differently.

**Conclusions:**

Assessing efficiency using the data that are available at the meso-level can serve as a first step in strengthening performance. Further work is required to support managers in the routine application of efficiency analysis and putting the results to use in guiding efforts to improve service delivery and increase utilization.

There is widespread consensus on the importance of strengthening health systems in low- and middle-income countries (LMIC) to redress health inequities and make the right to health a reality for vulnerable populations (1, 2). Service delivery is a crucial health system function for improving health and health equity, particularly in impoverished rural areas where great needs coincide with resource constraints (3). Strategies to strengthen service delivery in low-resource settings can take different approaches (4). One approach that is central to promoting health equity is advocating for increased resource allocation in under-served areas to expand access to health facilities, services, and goods. A complementary approach to strengthening services focuses on enhancing efficiency in utilization of existing resources to ensure that they are functioning to the best of their capacity with what is available so that the greatest health benefit possible may be attained.

Improving efficiency is important at all levels, but the potential impact in health gains is particularly great in services for the rural poor. Efficiency in the health sector means maximizing health gains obtained from a set of inputs, and the production processes that connect resource inputs to health gains occur in service delivery units (SDU) such as health posts (HPs), health centers, and hospitals. Measuring the efficiency of SDUs at the meso-level (district/regional) of the health system is useful for assessing and responding to variation in the performance of comparable SDUs (5). Existing studies of the efficiency of front-line rural health services in LMICs indicate that even under similar conditions within the same district or region, SDUs often have widely varying levels of efficiency (6–10). Results of such analyses can serve as a tool of formative evaluation for managers at the meso-level (11). By directing attention to SDUs at the high and low extremes of efficiency, it is possible to gain insight into what makes production processes work in their setting and focus supportive efforts where they are most needed (12).

Health services in rural Guatemala are delivered primarily through the public sector, and responsibility for planning, execution, supervision, and evaluation of health services and programs is held at the sub-national level of Regional Health Offices (*Dirección del Área de Salud*), whose jurisdictions correspond roughly to the 22 departments that make up the Republic of Guatemala. Health sector reforms initiated in the mid-1990s established the decentralization of administrative authority to the regional level in order to facilitate responsiveness to the needs and situations of the regions’ Municipal Districts which directly manage the provision of primary and secondary care services (13). Improving efficiency was among the principal objectives of the reform, along with increasing public spending on health and redressing inequities. However, institutional mechanisms for monitoring and evaluating efficiency were not established, and implementation of the decentralized model of administration has been limited by inadequately prepared health managers (14, 15).

Previous studies of the Guatemalan health system have highlighted different directions for strengthening performance. Inequitable access to services has been shown to be influenced by national as well as local issues. The magnitude and inequitable distribution of catastrophic spending due to ill health was demonstrated by Bowser and Mahal, who pointed to the need for stronger public financing mechanisms to reduce dependence on out-of-pocket spending (16). Health sector reform policy to expand coverage by contracting non-governmental organizations (NGO) to provide a basic package of services in the most remote rural areas has been reported to contribute to inequity through segmentation of the health system for different population groups (17). At a local level, access to and utilization of public health care services by rural indigenous families were found to be affected by the cost of transportation, linguistic and cultural barriers, and perceived disrespectful treatment (18). A recent study by Fort et al. suggested that expanded implementation of an inclusive model for primary health care designed for the rural Guatemalan context can contribute to strengthening performance, based on findings of improvement in utilization, quality and coverage over a 5-year period in two pilot sites (19). Efficiency has been examined in a previous study comparing different models of primary care provision (20). While these studies point to actions to improve performance at the national and micro-level, no studies aimed at enabling decision-making at the meso-level of the Guatemalan health system were found.

The assessment of the efficiency of public sector health services undertaken in this study provides an important complement to existing studies by indicating tools that are relevant for enabling regional managers to contribute to improving efficiency in the context of a decentralized administration. This study aims to measure the productivity of HPs in a rural department of Guatemala using data envelopment analysis (DEAs) to estimate technical efficiency (TE) and change in productivity during 2008–2009. The combination of analyses provides a more complete view of efficiency based on comparison to their peers and to their own efficiency the previous year. Findings will indicate the HPs at the high and low extremes of efficiency where regional managers can direct their efforts and gain insight into factors that facilitate and inhibit production processes in their context.

## Materials and methods

### The study site

This study was carried out in the department of Alta Verapaz located in the highlands of northern Guatemala, 200 km from the capital city. Alta Verapaz has 1.1 million inhabitants living predominantly in rural areas and 90% are indigenous, belonging to the Mayan ethnic groups Q'eqchi and Poqomchí. Agriculture is the main source of economic livelihood, including subsistence farming of beans and maize, and commercial farming of coffee and cardamom. Residents of Alta Verapaz have the highest rate of extreme poverty (38%) and the second highest rate of illiteracy (40%) in the country (21). Pneumonia, acute diarrheal diseases, and malnutrition are among the leading causes of mortality.

The Regional Health Office of Alta Verapaz is responsible for the administration and oversight of health programs and services in the department, and the units of Human Resources, Nursing and Statistics as well as the Director participated in the planning and development of this study. The region is divided into 19 Municipal Health Districts, with 17 health centers, and two district hospitals and one regional hospital that receive referrals. At the community level, primary care services are provided through 34 HPs and contracted NGOs. HPs tend to be located in larger villages or clusters of villages, while the contracted NGOs cover the most disperse and remote population using mobile health teams. The HPs typically cover a catchment area of around 2,000 inhabitants and are staffed by one to two auxiliary nurses whose work is supported by a team of community volunteers and supervised by a district nurse. They serve as a link between the community and the health system through health promotion activities, preventive and curative services, and referrals.

### Data envelopment analysis

The methodology of DEA has been applied extensively for analyzing the efficiency of primary health care SDUs in both high- and low-income countries (22). DEA is a non-parametric linear programming technique that allows comparisons across similar SDUs, which employ multiple inputs to produce multiple outputs. The TE of the units is calculated as the ratio of the weighted sum of outputs to the weighted sum of inputs, and unlike parametric techniques the method does not require mathematical specification of the production function relating outputs to inputs. The productions possibility frontier or ‘efficiency frontier’ is plotted based on the combinations of inputs and outputs from the best performing SDUs (23). The productivity of each unit is measured based on its distance from this frontier in order to determine its efficiency relative to the maximum level of efficiency observed in the sample. This results in the assignment of a TE score of 1 (100%) for units that compose the efficiency frontier and scores of less than 1 (0–99%) for units falling below the frontier.

Malmquist DEA methods allow for calculation of change in efficiency across two or more time periods. The analysis generates a total factor productivity change (TFPC) score, based in the geometric mean of two period productivity indices, in which 1=no change, <1=negative change, >1=positive change. The TFPC is decomposed into efficiency change and technical change to indicate the source of productivity change (24, 25). Efficiency change is change in a unit's production relative to the frontier with >1 meaning it is operating closer to the frontier than previously, and <1 indicating it is further from the frontier. Technical change means the frontier has shifted, possibly due to innovation or change in economic or regulatory policies. Monitoring change in productivity is useful for identifying SDUs that are improving or declining in the efficiency of their utilization of resources to attend health needs.

DEA has limitations that should be kept in mind when interpreting the results. The comparative analysis of the SDUs provides information about relative efficiency and the scores reflect how they compare to each other, based on the data entered. It is not possible to compare the SDUs’ productivity to an ideal standard (23). Variation is assumed to be due to differing levels of efficiency, but may in fact be due to other possible causes including epidemics, natural disasters, missing or erroneous data, or local socio-economic conditions. Thus, efficiency results are sensitive to outliers and should be interpreted with caution, particularly in the case of poor data availability. Also, because DEA utilizes a non-parametric function, it is difficult to apply statistical tests of hypothesis regarding possible factors associated with variation (24, 26).

### DEA variables

The offering of primary care services at the HP level is structured through ministry programs with a strong focus on maternal and child health priorities. These priorities and discussions with the regional director and the head of the Nursing Unit guided the collection of 15 output variables reflecting the priority health programs. Given the tendency of DEA to overestimate efficiency when the number of factors considered is relatively high, the number of outputs was limited to five with a balance of maternal, child, and general health services (24). Availability of data also influenced selection of the output variables. Service production data were readily available while data reflecting health promotion activities were more likely to be missing. The five output variables included: (1) number of new patients attended; (2) number of children less than two years old in growth monitoring; (3) number of prenatal follow-up visits; (4) number of children receiving a third dose of the DPT vaccine; and (5) number of family planning users.

The HPs are presumed to have similar inputs in terms of their physical structure and material resources. Information reflecting drugs and supplies allocated could be useful in analyzing efficiency. However, data available at the regional level only reflected resources allocated to the districts, not the individual HPs. The size of the population served by a HP is also presumed to be uniform, at around 2,000 inhabitants. Though in practice there is some variation in population served, census data from the catchment area of the HPs were not available at the regional level. Based on the data available, one input variable was used in the analysis: number of health workers, which in the case of HPs are auxiliary nurses.

### Data for DEA

Data on the outputs of each HP were collected for the years 2008 and 2009 from the national health information system (*SIGSA*) through the regional Statistics Unit. The data availability was fairly good, with 4% of values missing in 2008, and 2% in 2009. There were a few HPs with very low numbers for some output variables that suggested possible errors or poor reporting. However, the quality and availability of the data may also be considered as a reflection of how well the HP is working, so this was not considered a problem for the analysis.

There is not a similar national database registering the human resources utilized in the health services. Instead the information is managed at the regional and district levels. The input information for this analysis was collected from the registers of personnel maintained by the regional Human Resources Unit. For 2009, six of the 34 HPs were missing input values. However for 2008, the data availability was poor with 19 of 34 HPs missing input information. In those cases, the HPs were assigned a value of one health worker for 2008 and two health workers for 2009, based on the most common numbers of employees reported in HPs in those years respectively. Discussion with managers from the Human Resources Unit confirmed that this pattern was accurate because many new auxiliary nurses were hired to work in HPs in the last months of 2008 and beginning of 2009.

### Data analysis

In order to assess the efficiency of the HPs, the open-source software DEAP was used (27). After selecting appropriate input and output variables, there are a few considerations for creating the best model to enter into the software for analysis. The variables selected are tested for inter-correlation, so that variables that are correlated to each other may be narrowed down or combined in a composite variable. An inter-correlation test such as Spearman coefficient provides this information and a typical desired value to indicate non-correlation is greater than 0.6 (7, 10). The five output variables were tested for inter-correlation using STATA and were shown not to be inter-correlated.

Depending on whether there is more decision space or interest to exercise control over inputs or outputs, the DEAP software requires the user to specify whether the calculation of TE should be input or output oriented. In the case of primary and secondary care services, the inputs are usually few and are often fairly uniform across units yet the outputs may be increased through health promotion and outreach efforts. While in the case of hospitals offering primarily curative services with a large staff and variety of inputs, it may be more appropriate to adjust the balance of inputs than to influence the demand for curative services. It is also important to recognize that in many settings there is a great unmet need for health services and in such a situation, it would be unethical to recommend scaling down services, so output orientation may be the more appropriate choice (8). In the situation of HPs in Guatemala, an output-oriented analysis was done because at this level of care, the number of inputs (health workers) is largely dictated by the national level and there is greater potential to take action to increase the utilization of services, particularly through promotion of preventive services and improving patient satisfaction.

DEA can be based on assumptions of Constant Returns to Scale (CRS) or Variable Returns to Scale (VRS) across units. Under CRS, it is expected that units are operating at optimal scale and changes in input should generate a proportional change in outputs. While under VRS, it is assumed that all units may not be operating at their optimal scale and so their TE score is compared against other units of the same size. One component of the TE analysis comes from assessment of scale efficiency – that is whether the size of the facility is yielding outputs at the appropriate proportion. In the case of scale inefficiency, the unit may be exhibiting ‘increasing return to scale’ meaning it is too small for its scale of production or ‘decreasing return to scale’ meaning it is too large for the level of output it is producing. A VRS model was chosen because it was not expected that all SDUs were operating at optimal scale. Malmquist also requires specification of input or output orientation and CRS or VRS, and using the same criteria for the choice as with the TE assessment, output and VRS were selected.

As mentioned previously, DEA results are sensitive to outliers within the data set. While there were few missing values for HP outputs, the numbers reported varied widely. In order to assess the impact of outliers on the efficiency analysis, the jackknife technique was applied (28). The jackknife technique requires that efficiency scores are recalculated by dropping out units on the efficiency frontier (with a TE score of 100%) one by one. The similarity of the results of each recalculation to the results from the whole sample is estimated using Spearman rank correlation coefficients. A correlation value of 1 indicates that recalculated TE scores excluding a HP with 100% TE are the same as the results calculated from the whole sample, and thus the outlier does not influence the overall results. A value of 0 implies an absence of correlation, indicating that exclusion of the outlier completely changes the results. In this manner, jackknifing allows for assessment of the robustness of DEA results by estimating the influence of individual units on overall efficiency scores.

## Results

A total of 34 HPs were included in this analysis, and Table 1 shows descriptive statistics of the input and output data from 2008 and 2009. There is a wide variation in the outputs across the HPs as can be seen in the high values of the standard deviation in the outputs for both years. Also, there is a substantial increase between the mean production of 2008 and 2009, with three of the outputs increasing by more than 50%, while the average number of health workers per HP only increased by 36%.

**Table 1:** Descriptive statistics of health post outputs and inputs during 2008 and 2009

		1st visits	FP users	Child <2 yr in GM	Prenatal follow-up visits	3rd dose DPT	Health workers (input)
2008	Mean	2024.12	331.06	205.82	81.29	65.71	1.12
	Std Dev	1510.83	225.69	166.10	53.68	29.63	0.33
	(Min–Max)	(132–7276)	(7–1116)	(1–676)	(1–215)	(1–124)	(1–2)
2009	Mean	3071.44	443.65	480.38	147.5	79.26	1.74
	Std Dev	2156.47	231.03	345.59	75.17	35.08	0.62
	(Min–Max)	(345–11712)	(115–1158)	(1–1329)	(12–288)	(1–159)	(1–3)
2008–2009	% Change in Mean	+51.7%	+34.0%	+133.4%	+81.4%	+20.6%	+35.6%

DPT=diphtheria, pertussis, tetanus vaccine, FP= family planning, GM=growth monitoring.

**Fig. 1:**
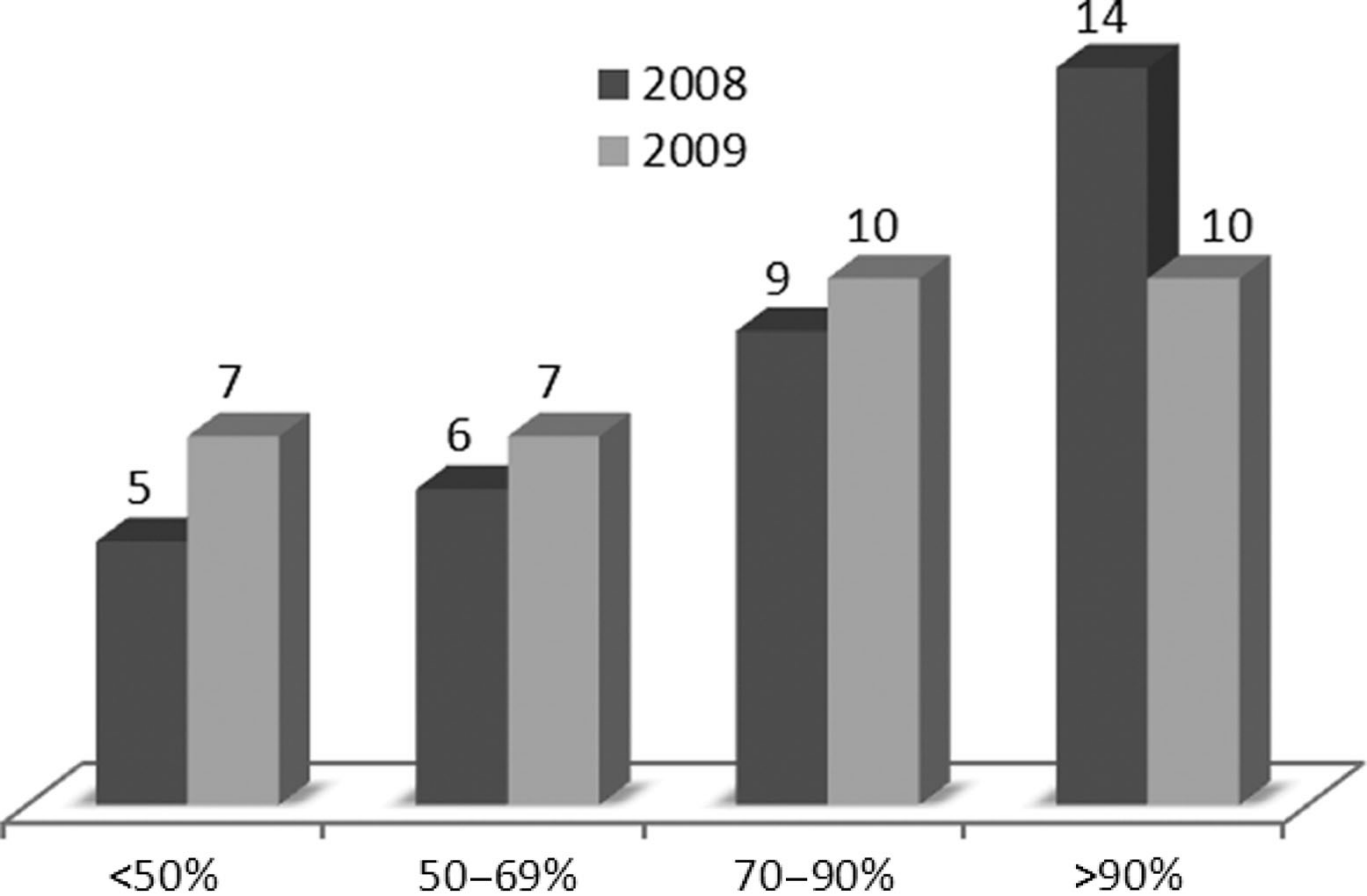
Distribution of technical efficiency scores 2008 and 2009. Number of HPs with TE scores within ranges representing high, moderate, poor and very poor efficiency.

The results of the TE analysis in 2008 and 2009 reveal a wide range of variation across the HPs during both 2008 and 2009 (Fig. 1 and Table 2). In 2008, 53% of the 34 HPs were operating with high efficiency, at or near the frontier of production (TE >90%), while 15% were operating with moderate efficiency (TE=70–90%), 21% with poor efficiency (TE=50–69%), and 15% with very poor efficiency (TE <50%). While in 2009, there was a decrease in the number of HPs operating with high efficiency: 29% were operating with high efficiency, 29% with moderate efficiency, 21% with poor, and 21% with very poor efficiency. This result indicates that the frontier of efficiency was determined by a smaller group of HPs in 2009, and the difference between their efficiency and the efficiency of their peers was more marked than in 2008. The results of the jackknife analysis indicated that the results were not affected by extreme outliers among the HPs which composed the efficiency frontier (TE=100%), as correlation coefficients ranged from 0.86 to 1.0.

**Table 2:** Technical efficiency (TE), Scale efficiency (SE) and Malmquist index scores for 2008 and 2009

	2008		2009		2008–2009		
			
Health post	TE (VRS)	SE	TE (VRS)	SE	Efficiency change	Technical change	TFP change
Sepoc	0.386	0.50 (drs)	0.451	0.56 (drs)	1.31	1.08	1.41
Cojaj	1.000	1	0.793	0.34 (drs)	0.27	1.49	0.40
San Agustin	0.948	1	1.000	0.56 (drs)	0.59	1.72	1.01
Chitocan	1.000	1	1.000	0.66 (drs)	0.66	1.07	0.70
Puribal	0.604	1	0.472	0.66 (drs)	0.52	1.01	0.52
Salacuim	0.791	1	0.734	0.50 (drs)	0.47	1.47	0.69
Saxoc	1.000	1	0.893	0.59 (drs)	0.53	1.12	0.59
Secopur	0.798	1	0.799	0.50 (drs)	0.50	1.62	0.81
Choval	0.725	1	0.859	0.38 (drs)	0.45	1.17	0.53
Bolonco	1.000	1	1.000	1	1.00	1.41	1.41
Chajmaic	0.978	1	1.000	0.53 (drs)	0.54	1.54	0.83
Tuila	0.880	1	0.675	0.52 (drs)	0.40	1.49	0.59
Nueva Palestina	0.511	1	0.574	1	1.12	1.76	1.97
Cahaboncito	1.000	1	0.995	0.51 (drs)	0.51	1.05	0.54
Rancho	1.000	1	1.000	0.69 (drs)	0.69	1.11	0.77
Santa Elena	0.495	1	0.465	0.61 (drs)	0.57	1.02	0.58
Campat	0.802	1	0.900	1	1.12	1.55	1.73
Chajaneb	1.000	1	0.661	1	0.66	1.88	1.24
Chamil	1.000	1	1.000	1	1.00	2.06	2.06
Chamisun	0.026	0.51 (drs)	0.213	0.50 (drs)	8.05	1.73	13.91
Saquil	0.666	1	0.369	1	0.55	2.08	1.16
Caquigual	1.000	0.58 (drs)	0.776	0.36 (drs)	0.33	2.07	0.68
Chacalte SJC	0.823	1	0.802	1	0.98	1.34	1.30
Pocola	0.381	0.50 (drs)	0.456	0.51 (drs)	1.22	1.61	1.95
Semesche	0.812	1	0.583	0.51 (drs)	0.37	1.61	0.60
Chijou	0.879	1	0.635	0.50 (drs)	0.36	1.49	0.54
Najquitob	0.586	1	0.827	0.62 (drs)	0.87	1.21	1.05
Actela	0.638	1	0.976	1	1.53	1.42	2.18
Camelias	0.464	1	0.670	1	1.44	1.36	1.96
Pasmolon	1.000	1	1.000	1	1.00	1.48	1.48
Chiacal	0.702	1	0.757	1	1.08	1.36	1.46
Chacalte T	0.594	1	0.476	0.51 (drs)	0.41	1.59	0.64
Cucanja	0.978	1	0.693	1	0.71	1.72	1.22
Raxquix	0.992	1	1.000	0.53 (drs)	0.53	1.90	1.01
Average score:	0.778		0.750		0.71	1.46	1.04

VRS=variable return to scales, drs=diminishing return to scales, TFP=total factor productivity.

Comparison of the scale efficiency scores of the HPs across the two years (SE columns, Table 2) indicated that the increase in outputs from 2008 to 2009 was not proportional to the increase in inputs. In 2008, 15 (44%) of the HPs were scale inefficient (SE<1), compared to 22 (65%) in 2009. All of these scale inefficient HPs exhibited diminishing returns to scale (drs), which indicates that their scale of production was less than should be expected based on their size (i.e. number of inputs).

The Malmquist productivity index allowed analysis of change in each HP's productivity from 2008 to 2009. The resulting TFPC indicates whether it has improved (TFPC >1) or deteriorated (<1) over time. Table 2 shows the results of the Malmquist index for each HP, including the TFPC and the efficiency and technical change scores. The total average TFPC score of 1.04 indicates that overall the HPs’ productivity increased by 4%. Of the 34 HPs, 53% experienced a positive change in productivity (TFPC>1) while 47% had a decline in productivity. HP Chamisun, which demonstrated an increase in productivity of 1,291%, was a notable outlier in this analysis. Its TE scores from 2008 and 2009 reflected poor performance compared to other HPs. However, jackknife analysis of results with and without HP Chamisun data indicated that its inclusion did not affect the efficiency frontier for either year.

The relative contributions of efficiency change and technical change to the TFPC for each SDU are shown in Table 2. The mean technical change score of 1.46 reflects changes in outputs causing a positive shift in the frontier, while the mean efficiency change (overall mean=0.71) indicates a decline in efficiency relative to the frontier from one year to the next. This means that the increase in TFP by 4% was due mainly to overall increases in outputs causing an outward shift in the frontier of efficiency. This increase in productivity may have been related to the implementation of a government program to fortify services in prioritized health regions during the end of 2008 and 2009, which included increases in numbers of auxiliary nurses as well as other staff at the district level (29). However, based on the short follow-up period after implementation and the limitations in the quality of the input data, it is not possible to draw conclusions about the program's influence on productivity.

**Fig. 2:**
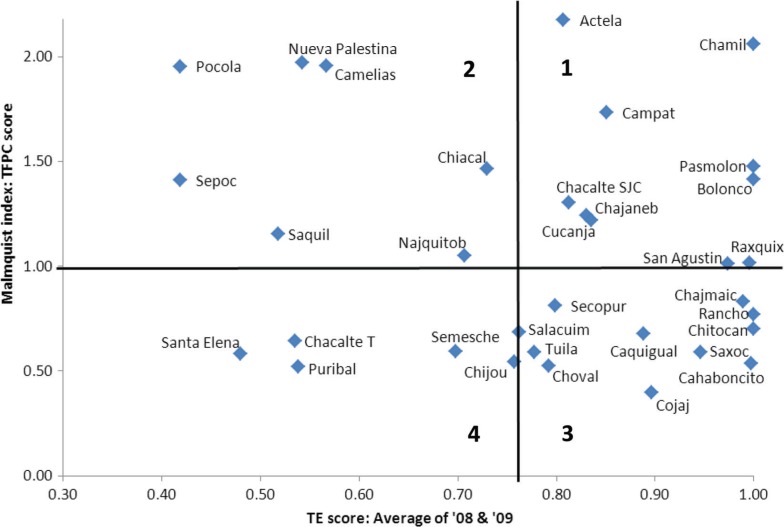
Bivariate plot of Technical efficiency (TE) and Total factor productivity change (TFPC) scores for each HP*. The plot is subdivided into four quadrants: 1=High TE, Improving TFPC; 2=Low TE, Improving TFPC; 3=High TE, Declining TFPC; 4=Low TE, Declining TFPC. *HP Chamisun (average TE=12.0, TFPC=13.9) is not shown to allow better visualization.

The combination of TE analysis and Malmquist analysis provides a more complete view of each HP's efficiency, based in comparison to their peers and their own efficiency over time. In order to facilitate visualization of patterns in the HPs’ performance and identify those at the high and low extremes of efficiency, the combined results are presented on an X–Y axis by plotting each HP based on its average TE score (2008 and 2009) as the X-coordinate and its TFPC score as the Y-coordinate (Fig. 2). The bivariate plot is divided into quadrants by a vertical line at the mean TE score of the HPs (0.78) and by a horizontal line at the TFPC score of 1.0, which represents no change in efficiency over the two years measured. Thus, the upper right quadrant (labeled 1) contains HPs with an above average TE score and a TFPC score reflecting increasing efficiency, and so may be considered High Efficiency, Improving. While the lower left quadrant (labeled 4) holds the HPs with a below average TE score in and a TFPC score reflecting a decrease in efficiency, and thus can be categorized as Low Efficiency, Declining. Likewise, the HPs in upper left quadrant (labeled 2) display Low Efficiency, Improving and the HPs in the lower right quadrant (labeled 3) have High Efficiency, Declining. This technique for presenting combined results provides management with a tool for detecting patterns across HPs so that efforts to improve efficiency can be directed to the low extremes where they are most needed, and local factors contributing to efficient processes at the high extremes can be identified and disseminated.

## Discussion

Assessing the efficiency of health service delivery in impoverished rural areas can serve as a first step to strengthening health system performance. This study provided two perspectives on HP efficiency in a rural department of Guatemala: TE and change in productivity over time. The TE scores of the HPs varied widely with 53 and 29% of HPs operating at or near the frontier (TE>90%) in 2008 and 2009, respectively. This indicated that despite facing similar resource-constrained conditions with similar inputs, some HPs are more successful in converting inputs to outputs than others. However, the rise in the number of HPs that were scale inefficient suggested that the increase in outputs was not proportionate to the increased number of inputs. The Malmquist index scores showed that while the overall mean total factor productivity increased by 4% from 2008 to 2009, 47% of HPs had a decline in productivity compared to the previous year. Combined results were presented in a bivariate scatter plot in order to facilitate observation of patterns and utilization of results to guide management efforts. Given the level of unmet need in this setting as well as the small number of health worker inputs per unit and the decision-making responsibilities accorded to the regional managers, these results are intended to guide efforts to increase utilization rather than evaluate resource allocation.

Application of DEA to assess efficiency of health care delivery in LMICs has increased in recent years, with the majority of studies conducted in Africa. However, there are fewer studies focused on primary health care provision at the community level. An early study on TE of peripheral health units in a district of Sierra Leone revealed an average TE score of 0.78 (SD=0.23), and 15% of units displayed very poor efficiency (TE<50%) (6). A more recent study in Sierra Leone found somewhat lower TE scores, with averages of 0.68 (SD=0.27), 0.69 (SD=0.33), and 0.59 (SD=0.35) reported for three different groups of units, and a greater proportion of units with very poor efficiency (22%, 32%, and 52%, respectively) (22). In the Tigray region of Ethiopia, HPs were found to have an average TE of 0.57 (SD=0.32) and 60% of units had very poor efficiency scores (7). While it is not possible to compare the true efficiency of SDUs across settings, because the TE scores are calculated in relation to the frontier of efficiency in each sample, it is noteworthy that for the years 2008 and 2009 Guatemalan HPs also displayed a wide dispersion of efficiency scores (SD=0.24 and 0.21). However, the average TE scores were somewhat higher (0.78 and 0.75) and there were fewer HPs with very poor efficiency scores (15 and 20%) compared to findings from the two African countries. Evaluation of change in the productivity of primary health care SDUs in a LMIC context with the Malmquist index was only found in one study in Seychelles (25).

This study contributes to an incipient but growing literature on efficiency analysis in Latin American health services. DEA studies conducted in Cuba and Mexico to assess the efficiency of urban health centers indicated higher TE scores with little dispersion, and few to zero units with very poor efficiency (TE<50%) (9, 10). A recent Chilean study applied DEA in a nation-wide comparative analysis of the efficiency of primary health care delivery at the municipal level, and found higher TE scores among urban than rural municipalities (30). In comparison, this study found relatively high levels of inefficiency in rural primary care services highlighting the importance of focusing research to strengthen health systems on regions where inequalities are greatest.

This study was limited by the data available reflecting inputs and outputs at the HP level. Human resources registers were incomplete, particularly for 2008. Efficiency scores for cases where the input estimate was incorrect may inaccurately depict their relation to their peers and their change in productivity over the two years. Including additional relevant inputs, such as supplies and capital resources, and using the population covered as a base to calculate staff per capita would have strengthened the analysis. However, these data were only available aggregated by district at the Regional Health Office, and not by individual SDUs. Data regarding specific inputs and population covered at the HP level are maintained in the districts and certainly have the potential to be gathered systematically at the regional level and included in national health information systems. Efforts to provide managers with analytical tools, such as DEA, to enhance data utilization in decision-making can provide impetus to improve data availability.

Availability of data for the selected outputs reflecting the quantity of services delivered under different priority programs was good. However, very low output values for some services from several HPs raise concern about data quality. Furthermore, while these outputs are a useful approximation, they do not fully capture the HPs’ contribution to health outcomes or the quality of the services delivered (31). Including data reflecting health indicators and user satisfaction as outcomes at the HP level would provide a more valid measure of the HPs’ efficiency in attaining health system goals.

While this study illuminated variations in efficiency across HPs, it did not provide insight into the causes of the variation. Several studies have applied a Tobit regression to analyze the correlation of DEA scores with environmental variables that may influence the SDUs’ production process, such as local socio-economic conditions and health worker characteristics (7, 30, 32).

Though additional data reflecting inputs, outcomes and environmental variables could have strengthened the analysis and indicated factors correlated with the variations in efficiency, this study utilized routinely collected data that are available at the regional level so that the efficiency assessment could be accessible as a monitoring tool for local managers. This was considered important so that efficiency analysis could potentially be incorporated into institutional practices at the regional health office. Even when data availability is limited, existing data can be better utilized to gain insight into variation in production processes across similar SDUs. Results provide managers with information that can help guide their efforts to improve efficiency by identifying units that are handling local conditions well and those in need of support.

## Conclusions

In order to strengthen the performance of primary health care services in vulnerable areas, regional managers need information about how well the units are utilizing the resources they receive. This study has shown how DEA methods can be applied at the meso-level of the health system to gain insight into variation in efficiency across primary health care SDUs and over time. The findings provided empirical evidence of the TE and productivity change of HPs in a rural, impoverished department of Guatemala over two years. These combined efficiency scores indicated which HPs are more and less efficient in delivering prioritized health services.

Further work is required to support regional managers in putting the results to use in enhancing efficiency. HPs identified at the high and low extremes of efficiency should be investigated further to determine how and why production processes are operating differently at these sites. Given the limited number and limited control over inputs at this level as well as the degree of unmet need in the region, efforts to enhance efficiency should focus on strategies to increase demand and utilization of services (outputs) rather than reduction of inputs (8). As managers gain insight into mechanisms promoting utilization in HPs with high efficiency, such as engagement with community leaders and quality of care, they can develop context-appropriate strategies for supporting HPs with low efficiency to improve their service and thereby better address unmet needs.
